# Point Values on Scoring Rubrics Influence Self-Regulated Learning for STEM Material

**DOI:** 10.3390/bs15040532

**Published:** 2025-04-15

**Authors:** Morgan D. Shumaker, Michelle L. Rivers, Sarah K. Tauber

**Affiliations:** 1 Department of Psychology, Texas Christian University, Fort Worth, TX 76129, USA; mrivers@scu.edu (M.L.R.); uma.tauber@tcu.edu (S.K.T.); 2 Department of Psychology, Santa Clara University, Santa Clara, CA 95053, USA

**Keywords:** self-regulated learning, value-directed remembering, rubrics, education

## Abstract

We examined whether point value information on a scoring rubric influences learners’ study time and concept selection when learning educationally relevant STEM information. Participants (*N* = 92) engaged in the self-regulated study of five concepts in mineral formation—geological processes, inorganic substances, compounds, elements, and crystalline solids—while having access to a scoring rubric that contained varying point values (concepts were worth 12, 8, or 4 points), uniform point values (all concepts were worth 8 points), or no point values for each concept. Participants chose how long to study and how many times to study each of the concepts. Concepts were selected for study more times when they were high-value than low-value on a grading rubric, an effect that was stronger for some concepts relative to others. Concepts were also studied slightly longer when they were high-value compared to low-value on a rubric. Our findings are consistent with value-directed remembering and agenda-based regulation and suggest that learners can use scoring rubrics to guide their decisions during learning.

## 1. Introduction

When studying for a course, students must self-regulate their learning by making decisions about what material to study and for how long to study it (Kornell & Bjork, 2007). Sometimes, instructors give students materials to help them guide their self-regulated learning such as a study guide or scoring rubric, which can make the instructors’ expectations for the assignment clear (Panadero et al., 2014). Students often aim to maximize their performance and, therefore, may use whatever resource their instructors gave them (e.g., a scoring rubric) to create an agenda by which to guide their self-regulated learning.

The agenda-based regulation framework posits that students assess task constraints prior to study and construct an agenda that allows them to achieve the task goal within those constraints (Ariel et al., 2009, 2015). Specifically, students use their agenda to select which items to study and to allocate study time to those items when self-regulating their learning (Ariel et al., 2015). Various factors can influence students’ agendas; one of which is the reward structure of the task. That is, students can construct an agenda aimed at maximizing their potential reward (Ariel et al., 2009).

One type of reward structure occurs when some of the material is deemed more valuable than other material (Middlebrooks & Castel, 2018). For example, an instructor might state that certain material will appear on a test, whereas other material (e.g., supplementary) will not. Prior work has found that when learning material that differs with respect to value, learners tend to spend more time studying (Soderstrom & McCabe, 2011) and choose to study (Middlebrooks & Castel, 2018) higher-value over lower-value information. Prioritizing high-value items over low-value items often leads to superior memory for the high-value compared to low-value items, an effect known as value-directed remembering (Knowlton & Castel, 2022; Castel et al., 2009, 2011, 2013).

If learners’ aim to maximize their score on an assignment where some material is worth more points than other material, focusing on high-value content would allow them to optimize their performance and achieve the task goal (Murphy & Castel, 2021b). In one study, when test items varied in point value (1 point or 5 points), participants more frequently chose to study the 5-point items, even though these items were more difficult to learn and likely required more time (Ariel et al., 2009). This suggests that students use the task’s reward structure to guide their study strategies, prioritizing higher-value items to self-regulate their learning.

Using point values to guide learning is consistent with both the agenda-based regulation framework and the value-directed remembering framework. Much research has investigated agenda-based regulation (Dunlosky et al., 2011) and value-directed remembering (for a review, see Knowlton & Castel, 2022). However, most research on value-directed remembering focuses on memory outcomes (e.g., Castel et al., 2009, 2011, 2013) and encoding mechanisms (Schwartz et al., 2023; Hennessee et al., 2019), and uses materials such as word lists (e.g., Middlebrooks & Castel, 2018; but see Murphy & Castel, 2021a; Friedman et al., 2015). Additionally, most research that examines agenda-based regulation uses materials such as word pairs (e.g., Ariel et al., 2009). Thus, little is known about how value influences learners’ study choices when engaging with educationally relevant materials, such as those encountered in STEM courses like geology.

One common scenario in which learners may encounter value-associated information is on a scoring rubric. To illustrate, instructors might provide a rubric to help students prepare a final research paper, indicating that the introduction section is worth 30 out of a possible 100 points on the assignment, whereas the method section is worth only 20 points. In this way, some components of the assignment carry more weight than others. This pedagogical strategy is presumably intended to direct students’ attention and effort toward concepts the instructor considers most important for developing content knowledge. From the agenda-based regulation perspective, a rubric should help shape learners’ study agendas and inform their study choices. If students aim to earn the highest possible score on the final paper, using the scoring rubric to prioritize high-value sections (e.g., the introduction) over low-value sections (e.g., the method) would be a strategic and beneficial approach.

Scoring rubrics can help learners better understand instructor expectations and support the development of more strategic study plans (Panadero et al., 2014; Reddy & Andrade, 2010). Additionally, giving learners access to a scoring rubric can increase their self-reported self-regulated learning (Camargo Salamanca et al., 2024). Specifically, learners who use rubrics while preparing for assignments report greater engagement in metacognitive processes such as the monitoring, planning, and evaluation of their learning compared to those without rubric access (Panadero & Romero, 2014). Learners’ self-reports also indicate that access to scoring rubrics enhances metacognitive engagement during study. Further, using rubrics as a self-assessment tool can foster better self-regulation, including increased use of learning strategies like memorization (Karaman, 2024). However, not all findings are consistent; some studies have found limited evidence that rubrics impact students’ self-regulated study behaviors (Fraile et al., 2023).

One limitation of previous research on learners’ rubric use is its heavy reliance on self-report measures, personal anecdotes (Panadero & Jonsson, 2020; Fraile et al., 2023), and varied experimental designs (Camargo Salamanca et al., 2024). Because self-reported study behavior can diverge from actual behavior, the degree to which scoring rubrics impact learners’ actual study choices remains an open question. Additionally, prior work differs with respect to the type of rubric used. Some rubrics contain subjective criteria (e.g., “all or most of the instruction involves active engagement”), others convey value-based information (e.g., “active engagement is worth 4 points”; Andrade & Du, 2005), and some provide scaffolded instructions (e.g., “underline key phrases in the rubric with colored ink”; Andrade et al., 2010; Andrade, 2019). Thus, a systematic examination of how point values on a rubric influence self-regulated learning has yet to be conducted. Specifically, to our knowledge, no study has compared rubrics with varying point values (high, medium, low) to rubrics with uniform (i.e., equally weighted) point values or rubrics with no point values. These latter formats may occur in practice when (1) an instructor indicates that all parts of the assignment are equally important and thus equally weighted, or (2) the instructor indicates the material that needs to be included but does not convey their relative point values.

The current study examines how point values on scoring rubrics influence self-regulated learning with objective measures of learners’ study choices when learning STEM material. Specifically, we examine how rubrics that differ in the point value information they provide affect two key behaviors: study time (the number of minutes learners spend reading a concept) and concept selection (the number of times a concept is chosen for study). Participants prepared for a writing assignment on mineral formation while viewing a rubric that indicated how many points each concept was worth on the assignment. Informed by the value-directed remembering framework, we hypothesized that high-value concepts would be chosen for study more times than lower-value concepts. We expected no difference in concept selection when concepts were associated with equal weight or no point values. Additionally, we expected that high-value concepts would be studied longer relative to lower-value concepts. Finally, we expected no differences in study time when concepts had no point values or if all concepts were weighted equally on the rubric.

## 2. Materials and Methods

This experiment was preregistered on the Open Science Framework (OSF) and can be accessed at https://osf.io/zua8s/?view_only=763e12df66184f8088121a83558477f8 (accessed on 27 March 2025).

### 2.1. Participants and Design

**Figure 1:**
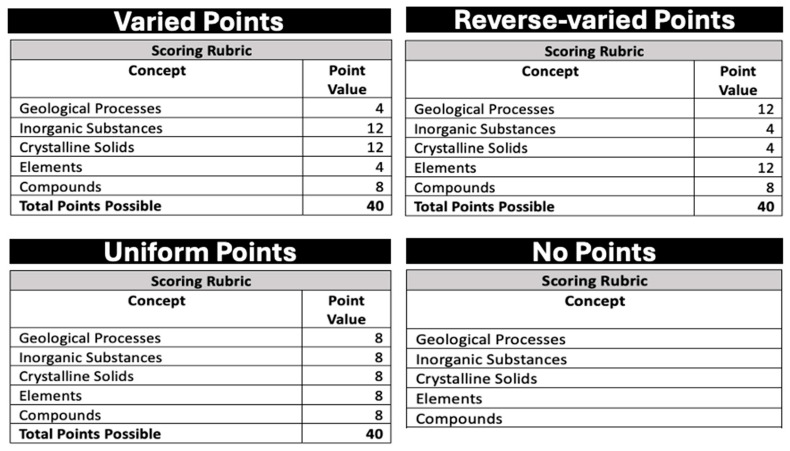
Rubric for each group.

Sample size was determined by conducting an a priori power analysis using G*Power 3.1.9.7 (Faul et al., 2007) to detect a medium effect (*η*^2^ = 0.06 or *f* = 0.25) with 95% power, an alpha error probability of 0.05, and a correlation among repeated measures of 0.20 in a mixed-model analysis of variance (repeated measures, within–between interaction) with four groups. The analysis revealed that we needed 136 participants (34 per group) to meet these criteria. Thus, data were collected from a total of 136 Texas Christian University undergraduate students who participated in the experiment for partial course credit. Participants’ ages ranged from 15 to 28 years old (*M* = 20.00, *SD* = 1.58) and most identified as women (20 men, 2 non-binary). Guardian consent as well as participant consent was gathered for participants under 18 years of age. Participants were randomly assigned to one of four rubric groups. Two rubric groups—uniform points and no points—were provided with the concepts that would appear on the writing assignment, but concepts were not differentiated in terms of point value (see bottom panel of Figure 1). The other two rubric groups—varied points and reverse-varied points—were provided with the concepts that would appear on the writing assignment and points values that varied between concepts (see top panel of Figure 1). The purpose of having two rubric groups was to counterbalance the concepts that were high-value and low-value. All participants studied the same five concepts about mineral formation (geological processes, inorganic substances, crystalline solids, elements, compounds). This research was approved by the Institutional Review Board at Texas Christian University.

### 2.2. Materials

Five STEM concepts in geology (geological processes, inorganic substances, crystalline solids, elements, and compounds) and the definitions for each were selected from the work of Ariel et al. (2021). Concept definitions were short paragraphs (~120 words) about how the concept relates to the process of mineral formation. Below is an example of the paragraph for geological processes:

“A mineral is a crystalline solid formed through geological processes. A geologic process is a natural process that occurs in or on Earth and that shapes Earth’s features, including volcanic activity; the movement of tectonic plates; the formation of sedimentary layers of sand and mud; and the folding of those layers until, deep in the Earth, they are exposed to high pressures and temperatures.”

We created four rubrics that differed by point value. In the groups with varied points and reverse-varied points, two concepts were assigned a high value (12 points), two concepts were assigned a low value (4 points), and one concept was assigned a medium value (8 points). In the group with uniform points, each of the five concepts was assigned a medium value (8 points). Finally, for the group with no points, each concept was listed without a point value (see Figure 1). Concepts were displayed on each rubric in a fixed order from top to bottom.

### 2.3. Procedure

This experiment was conducted in a lab, in groups ranging from one to eight participants who were tested simultaneously. As a measure of prior knowledge, participants were first asked to type everything they knew about each of the five concepts into a response box. Next, participants were instructed to learn about the process of mineral formation by reading about the individual concepts of mineral formation in preparation for a writing assignment similar to the one they had just completed (i.e., the prior knowledge test). Participants were also instructed to study as if they were preparing for an assignment in one of their classes and were informed that they would receive a rubric that includes each concept that would be on the final writing assignment. Then, participants were given an example rubric and informed that while studying, they could click on any of the five concepts, in any desired order, to read about them. Participants were informed that they had to study at least one concept but afterwards, they could either choose to move onto the writing assignment or continue studying the concepts as many times as desired.

Following the instructions, a rubric appeared at the top of the study screen with each concept listed as a button below in the same fixed order as on the rubric. To study a concept, participants clicked on the respective button to display a paragraph about how the concept related to mineral formation. Participants could spend as much time as desired reading about each concept. After studying a concept, participants clicked an arrow to move back to the study screen. On the study screen, participants were then given the option to continue studying any concept, in any order, for as long as desired or, to continue to the writing assignment.

**Figure 2:**
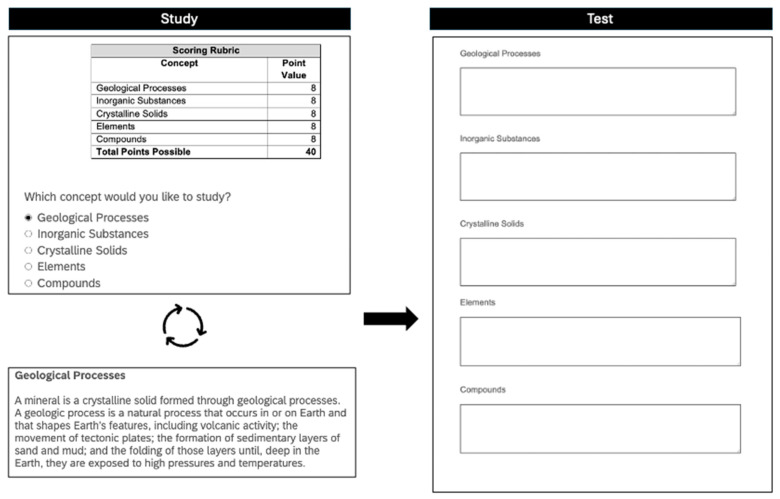
Task schematic. Participants began with the study screen, where they selected a concept to study (top left panel). They then read about the concept (bottom left panel) and returned to the study screen (top left panel) to make additional study selections or terminate study. Once participants terminated study, they completed the writing assignment (right panel).

Once participants were finished studying, they completed a writing assignment for which they were given the name of each of the five concepts of mineral formation and asked to type as much information as they could remember about each into a response box (see Figure 2). Participants wrote their answers in any order desired and took as much time as needed to complete the writing assignment; no feedback was provided.

Following the writing assignment, participants responded to the questions, “how did you determine that you were ready to complete the writing assignment?” and “did you use any strategies while preparing for the writing assignment?” by typing into a response box. Then, participants responded to the question, “how often do you get rubrics for writing assignments in your classes” and answered by selecting a multiple-choice option ranging from never to always. Participants also responded to the question “what is your opinion on having a rubric” and answered by selecting a multiple-choice option indicating that they like receiving a rubric, do not like receiving a rubric, or do not have a preference on receiving a rubric. Finally, participants provided demographic information and were debriefed about the goal of the study.

### 2.4. Data Scoring

Each geological definition was composed of multiple key idea units. For example, the concept geological processes contained three idea units, so participants could earn up to three points. One idea unit was “natural process that occurs in or on Earth” and students earned 0.5 points for “natural processes” and 0.5 points for “that occurs in or on Earth.” The second idea unit was “shapes Earth’s features” for which students could earn 1 point by stating that and could earn 0.5 points (half credit) for stating something like “shapes the planet.” For the third idea unit, participants could include “volcanic activity”, “movement of tectonic plates”, “formation of sedimentary layers of sand and mud”, or “folding of those layers until, deep in the Earth, when they are exposed to high pressures and temperatures” and could earn 1 point for listing any of those terms or phrases (i.e., participants did not need to list each term or phrase). The number of idea units ranged from three to five per concept definition (see OSF for the scoring rubric: https://osf.io/zua8s/?view_only=763e12df66184f8088121a83558477f8; accessed on 27 March 2025). Participants were not penalized for minor spelling errors (e.g., quarts instead of quartz). Two independent raters, masked to group assignment, scored all responses. For each concept, the raters were trained to assign no credit (0 points), partial credit (0.5), or full credit (1 point) to each idea unit based on the correctness and completeness of the response. Agreement between raters was high, *r* = 0.85, *p* < 0.001, so scores were averaged between the two raters.

## 3. Results

Data and analyses are available on the Open Science Framework at https://osf.io/zua8s/?view_only=763e12df66184f8088121a83558477f8 (accessed on 27 March 2025). Data were analyzed in *R* (version 4.1.2; R Core Team, 2022) using the lme4 (Bates et al., 2015) and lmerTest (Kuznetsova et al., 2017) packages. Bonferroni correction was applied to all follow-up analyses. We present the performance on concept selection, study time, short-answer test performance, and participants’ responses to post-task questions. Data are organized so that concepts can be compared between value conditions (high value, low value, uniform points, no points). Because the concept of compounds was constantly worth medium points (8 points), it could not be included in the analyses to evaluate the impact of different point values (i.e., high vs. low) on any outcome measure. We examine the effect of rubric value and concept on concept selection, study time, and short-answer performance.

### 3.1. Concept Selection

**Figure 3:**
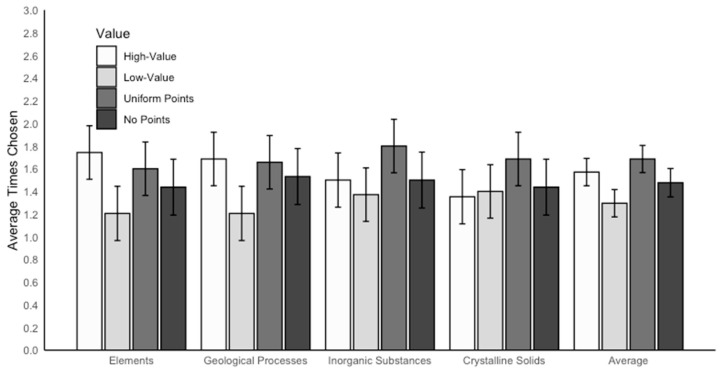
The average number of times each concept was chosen for study by rubric value. The concept of compounds is not included in this figure or associated analyses because it was used as a moderate-value control—i.e., it was not associated with either high or low values on any rubric.

We conducted a linear mixed effects model using rubric value and concept as interacting fixed effects and participant as a random effect to examine concept selection, defined as the number of times participants chose to study each concept. There was a significant main effect of rubric value on concept selection, *F*(3,169) = 8.07, *p* < 0.001, *η_p_*^2^ = 0.96, which is represented in the Average columns in Figure 3. Follow-up analyses revealed that concepts were selected for study significantly more often when associated with a high value (*M* = 1.57, *SE* = 0.07) compared with a low value on the rubric (*M* = 1.30, *SE* = 0.07), *p* < 0.001. In addition, concepts were selected for study more often when associated with the uniform points rubric (*M* = 1.69, *SE* = 0.09) compared with the low value on a rubric, *p* = 0.007. There was no effect of concept on concept selection, *F*(3,396) = 0.50, *p* = 0.683, and no interaction between value and concept, *F*(9,3577) = 0.84, *p* = 0.582.

Even though the interaction was not significant, we explored the effect of value on each concept separately to investigate the degree to which value impacted each. Linear regression models were conducted to examine the effect of rubric value on concept selection for each concept separately (see Figure 3). For elements, there was a main effect of rubric value on concept selection, *F*(3,132) = 3.74, *p* = 0.013, *η_p_*^2^ = 0.08. Follow-up analyses revealed that elements were selected for restudy significantly more often when associated with a high value (*M* = 1.74, *SE* = 0.12) relative to a low value on a rubric (*M* = 1.21, *SE* = 0.12), *p* = 0.011, *d* = 0.77. There were no other significant differences for the concept elements, *p*s ≥ 0.124. For geological processes, there was also a significant effect of rubric value on concept selection, *F*(3,132) = 3.48, *p* = 0.01, *η_p_*^2^ = 0.07. Follow-up analyses revealed that geological processes were selected for restudy significantly more often when associated with high value (*M* = 1.69, *SE* = 0.12) and uniform points (*M* = 1.66, *SE* = 0.12) relative to a low value on a rubric (*M* = 1.21, *SE* = 0.12), respectively, *p*s ≤ 0.045. There were no other significant differences for the concept geological processes, *p*s ≥ 0.348. There was no effect of rubric value on concept selection for inorganic substances, *F*(3,132) = 2.23, *p* = 0.087, or for crystalline solids, *F*(3,132) = 1.45, *p* = 0.230.

### 3.2. Study Time

**Figure 4:**
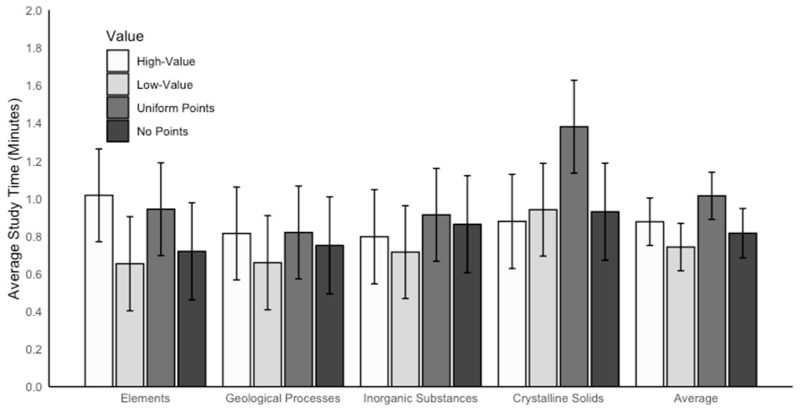
The average amount of time (in minutes) that each concept was studied by each rubric value. The concept of compounds is not included in this figure or associated analyses because it was used as a moderate-value control—i.e., it was not associated with either high or low values on any rubric.

Study time was recorded in Qualtrics as the total number of seconds participants spent on the study screen displaying information about each concept (see Figure 2) and was later converted to minutes. We conducted a linear mixed effects model using rubric value and concept as interacting fixed effects and participant as a random effect to examine study time. Outliers were considered those with study times more than two *SD* above the mean on any concept; there were 18 outliers. Excluding these outliers yielded similar patterns of results; thus, the full sample was maintained. There was a significant main effect of rubric value on study time, *F*(3,169) = 3.12, *p* = 0.028, *η_p_*^2^ = 0.90, which is represented in the Average columns in Figure 4. Post hoc comparisons revealed that high-value concepts were studied marginally longer (*M* = 0.88, *SE* = 0.08) than low-value concepts (*M* = 0.74, *SE* = 0.08), *p* = 0.059; however, this difference did not reach statistical significance. There were no other significant differences between rubric value groups on study time, *p*s ≥ 0.276. There was also a significant effect of concept on study time, *F*(3,396) = 10.19, *p* < 0.001, *η_p_*^2^ = 0.97. Participants studied crystalline solids (*M* = 1.03, *SE* = 0.06) for longer than geological processes (*M* = 0.76, SE = 0.06), inorganic substances (*M* = 0.82, *SE* = 0.06), and elements (*M* = 0.83, *SE* = 0.06), *p*s ≤ 0.004. There were no other significant differences between concepts on study time, *p*s > 0.999.

Importantly, these effects were qualified by a significant interaction between rubric value and concept, *F*(9,338) = 2.20, *p* = 0.021.

The interaction was driven by study time differences for one concept—crystalline solids. Indeed, follow-up linear regression models revealed a marginal effect of rubric value on study time for crystalline solids, such that crystalline solids were studied longer when associated with uniform points (*M* = 1.38, *SE* = 0.15) compared with a low value on a rubric (*M* = 0.94, *SE* = 0.15), *F*(3,132) = 2.54, *p* = 0.058 (see Figure 4); this trend was not significant. There was no effect of rubric value on study time for elements: *F*(3,132) = 1.67, *p* = 0.176. There was also no effect of rubric value on study time for geological processes, *F*(3,132) = 0.44, *p* = 0.722, or for inorganic substances, *F*(3,132) = 0.65, *p* = 0.586.

### 3.3. Short-Answer Test Performance

**Figure 5:**
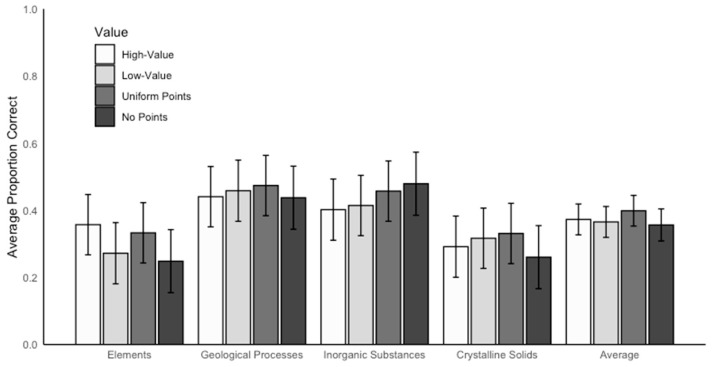
The average proportion correct on the short-answer test for each concept by rubric value. The concept of compounds is not included in this figure or associated analyses because it was used as a moderate-value control—i.e., it was not associated with either high or low values on any rubric.

We conducted a linear mixed effects model using rubric value and concept as interacting fixed effects and participant as a random effect to examine short-answer performance, defined as the correct proportion on the short-answer test. There was no effect of rubric value on short-answer performance, *F*(3,169) = 0.29, *p* = 0.832 (see the Average columns in Figure 5). However, there was a significant main effect of concept, *F*(3,396) = 23.07, *p* < 0.001, *η_p_*^2^ = 0.99 (see the Concept columns in Figure 5). Post hoc comparisons revealed that participants performed better on geological processes (*M* = 0.45, *SE* = 0.02) and inorganic substances (*M* = 0.44, *SE* = 0.02) than on crystalline solids (*M* = 0.30, *SE* = 0.02), *p*s < 0.001, *d*s ≥ 0.68. Additionally, participants performed better on geological processes and inorganic substances than on elements (*M* = 0.30, *SE* = 0.02), *p*s < 0.001, *d*s ≥ 0.67. There were no other significant differences between concepts on short-answer performance, *p*s > 0.999.

We also conducted an exploratory moderation analysis to examine whether the relationship between rubric value and short-answer performance was moderated by concept selection. The overall model accounted for a significant portion of the variance in short-answer performance, *F*(7,536) = 5.56, *p* < 0.001, *R*^2^ = 0.07. More frequent concept selection was associated with higher short-answer performance when no points were assigned to concepts, *b* = 0.08 (*SE* = 0.03), *t* = 2.52, *p* = 0.012. This relationship was not present when concepts were associated with high, low, or uniform point values, *p*s ≥ 0.725. Overall, concept selection did not moderate the effect of rubric value on short-answer performance, *p*s ≥ 0.400.

Finally, we conducted an exploratory moderation analysis to examine whether the relationship between rubric value and short-answer performance was moderated by study time. The overall model accounted for a significant portion of the variance in short-answer performance, *F*(7,536) = 14.74, *p* < 0.001, *R*^2^ = 0.16. When no points were assigned to concepts, increased study time was associated with better short-answer performance, *b* = 0.10 (*SE* = 0.03), *t* = 3.19, *p* = 0.002. This relationship was not significant when concepts were assigned uniform points, *b* = −0.04 (*SE* = 0.04), *t* = 0.99, *p* = 0.320. However, study time was a significantly stronger predictor of short-answer performance when concepts were assigned high values, *b* = 0.12 (*SE* = 0.04), *t* = 2.69, *p* = 0.007, and when concepts were assigned low values, *b* = 0.13 (*SE* = 0.05), *t* = 2.65, *p* = 0.008, than when concepts were assigned no points. Thus, study time significantly moderated the relationship between rubric value and short-answer performance.

### 3.4. Responses to Post-Task Questions

Responses to “how did you determine that you were ready to complete the writing assignment” and “did you use any strategies while preparing for the writing assignment” were coded according to a scoring rubric. Many participants (47%) indicated that they determined they were ready to complete the writing assignment when they felt their recall was sufficient. Fewer participants reported that they decided they were ready for the test after they had read all the materials at least once (25%), when they felt confident about their knowledge (21%), when they could not remember any more information (4%), other (2%), or that they never felt ready (2%). There were no differences between rubric groups on how participants determined they were ready for the final writing assignment, *χ*^2^(15) = 9.66, *p* = 0.840.

Additionally, many participants (51%) reported that they used multiple strategies (e.g., retrieval practice and key words) while preparing for the writing assignment. Fewer participants reported that they did not use any particular strategy (10%), or that they used one strategy. Specifically, some participants reported that they used repetition (10%), memorization (9%), key word or main idea identification (4%), connections to other material (4%), retrieval practice (3%), what they did not know (3%), mnemonics (2%), exclusively the point values on the rubric (1%), or another strategy such as chunking (4%). There were no differences between rubric groups on their self-reported strategy use, *χ*^2^(30) = 20.10, *p* = 0.915.

There was inconsistency in participants’ reports of how often they receive rubrics in their classes. Some participants indicated that they receive rubrics for writing assignments in their actual classes (18% always receive rubrics; 24% sometimes receive rubrics), whereas other participants indicated they do not (8% do not often receive rubrics; 3% never receive rubrics). There were no differences between rubric groups on the frequency of receiving a rubric, *χ*^2^(12) = 18.00, *p* = 0.114. Most participants indicated that they like having a rubric (91%), and very few participants reported no preference on receiving a rubric (7%) or that they do not like receiving a rubric (2%). There were no differences between rubric groups on preference for receiving a rubric, *χ*^2^(6) = 6.50, *p* = 0.369.

## 4. Discussion

We examined the effect of scoring rubrics on learners’ concept selection and study time when learning educationally relevant STEM material. Learners studied various concepts about mineral formation while having access to a rubric that indicated each concept was worth a different number of points or that all concepts were worth an equal number of points or a rubric that did not convey any information about point value for the concepts. Participants whose rubrics indicated that elements and geological processes were high-value chose to study those concepts more times than those whose rubrics indicated those concepts were low-value. Additionally, when studying geological processes, those in the uniform points (medium-value) group studied more times than those in the low-value group. There was also a trend such that when concepts were deemed high-value, participants studied numerically longer than when concepts were deemed low-value. There were no differences in concept selection or study time between those whose rubrics indicated that each concept was equally weighted or whose rubrics did not contain value. Thus, there is some evidence that learners made strategic choices (albeit imperfectly) based on the concept value when point values differed between concepts (high- vs. low-value). These outcomes are consistent with the value-directed remembering framework (Knowlton & Castel, 2022; Castel et al., 2009, 2011, 2013).

These findings are also in line with the agenda-based regulation framework (Ariel et al., 2009), suggesting that when concepts differed by point value on the rubric, learners used that information, at least to some extent, to create their agendas to meet their goal of maximizing their scores on the assignment. That is, when information on the rubric was useful (i.e., when concepts differed by point value), learners used the point values on the rubric to guide their concept selection (although, interestingly, many of them did not report doing so). This is, to our knowledge, the first objective evidence that learners do indeed use scoring rubrics as a basis for their study behavior when anticipating a writing assignment. Moreover, most learners report that they like having a rubric when completing an assignment. Future research could examine which types of rubrics best support students’ self-regulated learning, as the rubrics in this experiment differ from rubrics used in prior work (e.g., Andrade, 2019). The diversity in educators’ approaches to rubric construction may contribute to interpretation challenges for learners and may directly impact the degree to which learners effectively use rubrics to direct their learning behavior.

Test performance was not influenced by value. This finding is inconsistent with prior work in value-directed remembering that has shown better memory for higher-value than lower-value material (e.g., Knowlton & Castel, 2022). This outcome is important for contextualizing the educational implications of the reported experiment because this suggests that even though value from the rubric influenced some of learners’ study decisions, it did not have a meaningful impact on test performance. However, there are multiple reasons why we did not observe test performance effects. One reason may be that in typical value-directed remembering paradigms, learners need task experience (e.g., multiple trials) to learn to prioritize high-value over low-value information (e.g., Castel et al., 2009, 2011, 2013). Participants in our study only had one instance of having a scoring rubric while completing an assignment, so it will be important for future work to examine whether learners need practice using rubrics to use them more effectively. A second reason is that even though learners chose to study high-value concepts more times than other concepts, they may have needed to revisit concepts more times, spend more time with them, and use more effective learning strategies when learning the high-value concepts to observe test performance effects. A critical direction for the future will be to build on the reported outcomes to determine how to increase the quality of learners’ study during self-regulated learning. One approach would be to conduct a classroom study in which students are more motivated to learn content that is directly needed for their class. In this context, value effects from a provided rubric may appear on test performance for the learners’ class. At present, we recommend that educators provide rubrics that differentiate concepts by value so students can direct their study in preparation for class assignments and exams.

Additionally, the current study did not assess delayed test performance, which would be important both from the perspective of value-directed remembering effects and practical applications of the current research. Future work should examine how value portrayed on a rubric influences lasting learning. Finally, concept selection did not moderate the relationship between value and short-answer performance. However, increased study time was associated with better short-answer performance when concepts were associated with no points, and this relationship was even stronger when concepts were associated with high value sand low values. This suggests that students may use study time more strategically when rubrics emphasize certain concepts (e.g., high-value or low-value) over others (e.g., uniform points).

### 4.1. Limitations

This study is not without its limitations. First, the finding that learners selected to study concepts more times when they were high-value compared to low-value was driven primarily by some concepts rather than all concepts. This finding indicates that considering item characteristics will be critical for determining when and why learners’ study decisions are influenced by value. Future research is needed to systematically evaluate the characteristics of complicated concepts (e.g., interest, applicability, abstractness) like the mineral formation concepts used in the reported experiment to determine what factors influence self-regulated learning when students have value information provided on a rubric. For instance, learners’ study time may have been influenced by item difficulty, as indicated by differences in short-answer test performance. Specifically, crystalline solids was one of the more challenging concepts with lower test performance relative to geological processes and inorganic substances (*p*s ≤ 0.001).

Another limitation is that learners’ study decisions may have also been influenced by habitual responding (e.g., studying from top to bottom; Dunlosky & Ariel, 2011). That is, concepts appeared in a fixed order on each rubric and on the concept selection screen for all participants. We adopted this method because it reflects common instructional practices. However, we suggest that follow-up research randomize the order of concepts on rubrics. This is important given that we found value effects to be stronger on learners’ study choices for some concepts relative to others. Given that participants performed best on the first two listed concepts (geological processes and inorganic substances), it is possible that they better remembered these concepts simply because they studied them first. Indeed, exploratory analyses found that 67.65% of participants studied geological processes (the first listed concept) first. Further research is needed to disentangle the effects of habitual responding, learners’ preferences for content, and value on short-answer performance.

### 4.2. Conclusions

Most importantly, we found evidence that learners can use rubrics when self-regulating their learning of complicated STEM concepts. Scoring rubrics that provide learners with information about value differences between concepts can influence learners’ studying behavior in a laboratory setting. Future research could expand on these findings by examining whether scoring rubrics that differ with respect to value influence learners’ studying behavior in a classroom setting, as students may be more motivated to use the scoring rubric to guide their learning. The current study also provides some evidence for value-directed and agenda-based regulation effects using educationally relevant material such as a scoring rubric. Scoring rubrics that contain information regarding how many points each topic is worth on an assignment may be beneficial for students’ self-regulated learning. These findings have practical implications, as instructors could provide scoring rubrics where the material varies in point value to help students self-regulate their learning by guiding them toward prioritizing high-value over low-value information.

## Data Availability

All materials, raw data, and analysis code are available from the Open Science Framework and can be freely accessed at https://osf.io/zua8s/?view_only=763e12df66184f8088121a83558477f8 (accessed on 27 March 2025).
